# Fecal Incontinence after Posterior Sagittal Anorectoplasty for Anorectal Malformation: A Single-Center Study

**DOI:** 10.1155/2018/8297617

**Published:** 2018-05-30

**Authors:** Manoochehr Ghorbanpoor, Behzad Dehvan, Siavash Rahimi, Azar Pirdehghan

**Affiliations:** ^1^Department of Surgery, Hamadan University of Medical Sciences, Hamadan, Iran; ^2^School of Medicine, Mazandaran University of Medical Sciences, Sari, Iran; ^3^Department of Community and Preventive Medicine, School of Medicine, Hamadan University of Medical Sciences, Hamadan, Iran

## Abstract

**Background:**

Fecal incontinence is one of the worst functional complications of posterior sagittal anorectoplasty for treatment of anorectal malformation.

**Objectives:**

In this study, we aimed to identify the prevalence of fecal incontinence in patients with the diagnosis of high or low anorectal malformation who underwent three-stage posterior sagittal anorectoplasty surgery in our center.

**Patients and Methods:**

Children with the diagnosis of anorectal malformation who underwent posterior sagittal anorectoplasty at the Department of Pediatric Surgery of Besat Hospital, Hamadan University of Medical Sciences, Iran, from 2012 to 2016 were enrolled in the study. Parents or guardians were recruited and asked to fill the study questionnaire including the Templeton and Ditesheim Scoring System to assess the status of fecal continence of the patients.

**Results:**

Thirty-four patients including 10 (29.4%) males were enrolled in the study. High type of anorectal malformation was diagnosed in 23 (67.6%) patients. The overall mean scores of fecal continence were 4.57 ± 0.84 (range 1.5–5) after a mean follow-up time of 50.7 (range 22.5–69.8) months. Good fecal continence was observed in 91.3% of patients with low type compared to 72.8% of patients with high type of anorectal malformation; however, the difference was not significant (*P*=0.13).

**Conclusion:**

Posterior sagittal anorectoplasty surgery in patients with anorectal malformation may result in acceptable fecal continence.

## 1. Introduction

Anorectal malformation characterizes as a spectrum of congenital abnormalities where the anus fails to open normally onto the perineum classified as low and high types based on the relationship of the terminal colon to the levator muscles of the pelvic floor. It is considered the most frequently encountered anomaly in neonatal pediatric surgery which occurs approximately 1 in 5000 live births [1]. Despite recent advancement of surgical techniques, treatment of anorectal malformation is still a difficult challenge for pediatric surgeons.

Posterior sagittal anorectoplasty initially introduced by deVries and Pena in 1982 has been currently considered as the standard surgical treatment of choice for anorectal malformation worldwide [2]. However, the challenge regarding its long-term outcome and prognosis remains. Surgical complications included bowel incontinence, soiling, and constipation with negatively affected quality of life and daily activities in such patients [3]. Fecal incontinence is one of the worst functional outcomes of this procedure with a reported incidence rate of 22–65% based on different continence scoring systems [4–7].

In this study, we aimed to identify the prevalence of fecal incontinence in patients with the diagnosis of high or low anorectal malformation who were treated with three-stage posterior sagittal anorectoplasty surgery in our center.

## 2. Patients and Methods

### 2.1. Patient Selection

The operative registry of patients with the diagnosis of anorectal malformation who underwent three-stage posterior sagittal anorectoplasty at the Department of Pediatric Surgery of Besat Hospital, Hamadan University of Medical Sciences, Iran, from 2012 to 2016 was retrospectively reviewed. Inclusion criteria consisted of patients who underwent posterior sagittal anorectoplasty for treatment of anorectal malformation. Patients with mental retardation, neurological disorders, concomitant anomalies, and history of other anorectal surgeries were excluded from the study. Also, one patient with low type of anorectal malformation who underwent just anoplasty surgery was excluded from the study.

### 2.2. Surgery

All live newborns who failed to pass meconium within the first two days of life with no anal opening visible underwent three-stage operation. Initially, a double-barrel colostomy was performed within 48 hours of life. Four weeks later, a distal colostogram was performed to identify the anatomy of the distal bowel and the type of anorectal malformation. Then, the second surgical procedure was performed when the patients gained normal weight or above for age based on the World Health Organization standard growth charts. A longitudinal incision from coccyx to the perineal body is made to uncover the external anal sphincter, levator ani muscle, rectum, and distal fistula. Then, separation of the fistula from the urethra or vagina was performed, and the rectum was sutured to the anus after reconstruction of the perineum. In a postoperative visit at two weeks after the second operation, dilation of the anorectal site was performed to avoid stenosis and was continued daily by the parents for a month. Thirdly, closure of the colostomy was performed at four weeks after anorectoplasty. All surgeries were performed by a same surgeon under general anesthesia. At every stage of the surgical operation, intravenous antibiotics were given and were continued for 72 hours after the operation. Feeding was gradually reintroduced when bowel function resumed postoperatively.

### 2.3. Study Protocol

Patients and their parents or guardians were recruited, and data of the patients about demographics, the type of anorectal malformation, and the status of fecal continence using the Templeton and Ditesheim Scoring System shown in Table 1 were recorded [8]. Based on the aforementioned scoring system, patients with 4-5, 2–3.5, or 0–1.5 points were considered as having good, fair, or poor fecal continence, respectively. The study was conducted after approval of the Ethical Committee of Hamadan University of Medical Sciences and taking an informed consent from the parents or the guardians of the patients.

### 2.4. Statistical Analysis

Statistical analyses were performed using the SPSS software version 22.0 for Windows (SPSS Inc., Chicago, IL). Qualitative and quantitative data were compared between the two groups using the Fisher exact test and the independent *t*-test, respectively. A *P* value of less than 0.05 was considered statistically significant.

## 3. Results

Thirty-four patients including 10 (29.4%) males were enrolled in the study. Low anorectal malformation was diagnosed in 23 (67.6%) patients. Concomitant genitourinary or skeletal anomalies were found in 16 (47%) patients including two with defects in both systems. Genitourinary anomalies were found in 10 (29.4%) patients including three with vesicoureteral reflux, two with neurogenic bladder, two with vaginal agenesis, two with single kidney, and one with horseshoe kidney. Skeletal anomalies were also found in eight (23.5%) patients including four with vertebral malformation, three with sacral agenesis, and one with radium agenesis.

**Figure 1:**
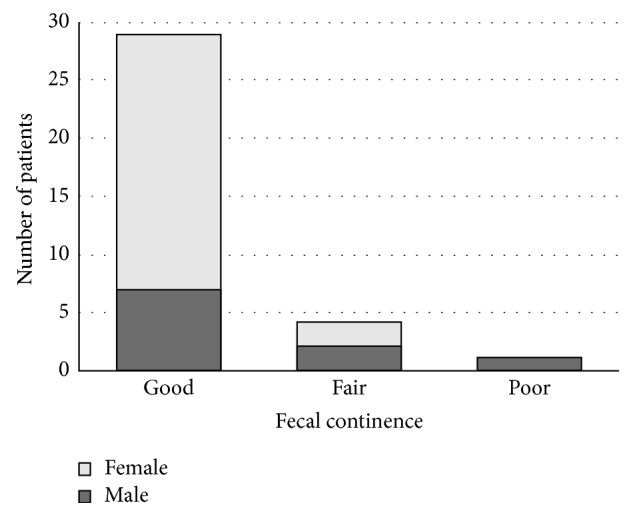
Number of patients with different statuses of fecal continence and their gender distribution.

The overall mean score of fecal continence was 4.57 ±0.84 (range 1.5–5), and 29 (85.3%) patients maintained good fecal continence after a mean follow-up time of 50.7 (range 22.5–69.8) months after the definite repair surgery. Number of patients with different statuses of fecal continence and their gender distribution are shown in Figure 1. Good fecal continence was observed in 91.7% of female patients compared to 70% of males; however, the difference was not significant (*P*=0.16). Besides, good fecal continence was determined in 91.3% of patients with low type compared to 72.8% of patients with high type of anorectal malformation; however, the difference was not significant either (*P*=0.13).

Associated anomalies were found in 80% of males compared to 33.3% of females and in 81.2% of patients with high type of anorectal malformation compared to 30.4% with low type of anorectal malformation (*P*=0.023  and  *P*=0.009, resp.). Good fecal continence was observed in 81.2% of patients with associated anomalies compared to 88.9% of patients without anomalies; however, the difference was not significant (*P*=0.64).

Patients with good, fair, and poor fecal continence underwent posterior sagittal anorectoplasty surgery at mean ages of 2.76 ± 3.6, 1.75 ± 1.5, and 1 ± 0 months, respectively (*P*=0.77), and were evaluated for fecal continence at means of 51.8 ± 29, 52.5 ± 36.5, and 48 months, respectively (*P*=0.99).

## 4. Discussion

In our study, the overall prevalence of fecal incontinence was 14.7%. Although the rate of good fecal continence was higher in females compared to males, patients with low type compared to high type of anorectal malformation, and patients without associated anomalies compared to the ones with associated anomalies, the observed differences were not significant. Studies have reported a fecal incontinence rate of 30–50% in children who underwent posterior sagittal anorectoplasty for treatment of anorectal malformation. In a study by Kubota et al., 41% of patients who underwent posterior sagittal anorectoplasty experienced fecal incontinence after 3 years of follow-up using the Japan Study Group of Anorectal Anomalies scoring system [9]. In a study by Huang et al., 188 children including 85 with low anorectal malformation and 103 with high anorectal malformation were retrospectively studied. After 4.3 years of follow-up, fecal incontinence occurred in 4.7% and 3.9% of patients with the low and high anorectal malformation, respectively, with no significant difference [10]. Askarpour et al. reported that 31.7% of 60 patients after posterior sagittal anorectoplasty experienced fecal incontinence using the Rintala scoring system with no significant difference regarding the type of anorectal malformation, age, and gender of the patients [11]. In a study by Stenstrom et al., fecal incontinence was reported in 42% of males and 48% of females with no difference after a median follow-up of 8 years using the Krickenbeck classification [12]. In contrast, in a study of Borg et al., fecal incontinence after 10 years of follow-up was reported in 64% of males compared to 20% of females with a significant difference [13].

The most important reason for the observed differences through the various studies may be using the different fecal continence scoring systems which assess the bowel function of the patients through different aspects providing different answering options. The Templeton and Ditesheim Scoring System used in our study is a quantitative method which has been shown to be comparable with Kelly [14], Kiesewetter [15], and Wingspread [16] fecal continence scoring systems; however, a higher continence score assignment has been reported than the others [17]. Over time, different scoring systems including Holschneider [18], Rintala [19], Krickenbeck [20], and Pena [21] for analyzing the bowel function have been proposed; however, there are great variations between their results. It has been shown that using Krickenbeck and Pena questionnaires in patients with low type of anorectal malformation may result in a lower score assignment compared to the patients with high type of anorectal malformation while using Holschneider and Rintala questionnaires [22]. Hence, designing a standard unified questionnaire for assessing bowel function in patients with anorectal malformation is highly desirable.

Another rationale for the different results may be the age difference of the studied populations. Some studies like ours reported that there was no association between the age of the patients and functional outcomes after posterior sagittal anorectoplasty, whereas some others stated that bowel function may improve with age [11, 23]. This difference may be explained by the fact that older patients are more mindful of themselves and more confident to participate actively in all aspects of their lives; however, it may also be due to the intrinsic differences of the various questionnaires.

## 5. Conclusion

Our study may suggest that the postsagittal anorectoplasty surgery in patients with anorectal malformation may result in acceptable fecal continence. However, data might be more definite if there is a standard unified scoring system through various studies.

## Figures and Tables

**Table 1:** Templeton and Ditesheim Scoring System for assessing fecal continence.

	Point
*Toilet training for stool*	
Successful	1
Occasionally successful (awareness of impending stool)	0.5
No awareness of impending stool	0
*Accidents*	
None or rare	1
Three times per week or less	0.5
More than three times per week	0
*Extra underpants or liners needed*	
Never	1
Only when having diarrhea	0.5
Always	0
*Social problems*	
None	1
Infrequent order: does not miss school, but no overnights, dates, and camping	0.5
Frequent order affects school and play	0
*Activity restrictions*	
None	0.5
Avoids swimming, sports	0
*Rashes*	
No current problems	0.5
Some current problems	0

## Data Availability

The data used to support the findings of this study are available from the corresponding author upon request.

## References

[1] Levitt M. A., Pena A. (2005). Outcomes from the correction of anorectal malformations. *Current Opinion in Pediatrics*.

[2] deVries P. A., Pena A. (1982). Posterior sagittal anorectoplasty. *Journal of Pediatric Surgery*.

[3] Rintala R. J., Pakarinen M. P. (2008). Imperforate anus: long- and short-term outcome. *Seminars in Pediatric Surgery*.

[4] Langemeijer R. A. T. M., Molenaar J. C. (1991). Continence after posterior sagittal anorectoplasty. *Journal of Pediatric Surgery*.

[5] Davies M. C., Creighton S. M., Wilcox D. T. (2004). Long-term outcomes of anorectal malformations. *Pediatric Surgery International*.

[6] Bukarica S., Marinkovic S., Pekovic-Zrnic V., Dobanovacki D., Borisev V., Likic J. (2004). Clinical evaluation of fecal continence after posterior sagittal anorectoplasty in anorectal abnormalities. *Medicinski Pregled*.

[7] Rintala R. J., Lindahl H. (1995). Is normal bowel function possible after repair of intermediate and high anorectal malformations?. *Journal of Pediatric Surgery*.

[8] Templeton J. M., Ditesheim J. A. (1985). High imperforate anus–quantitative results of long-term fecal continence. *Journal of Pediatric Surgery*.

[9] Kubota A., Nara K., Kawahara H. (2011). Therapeutic strategy for persistent cloaca: the efficacy of antegrade continent enema as a salvage surgery. *Pediatric Surgery International*.

[10] Huang C.-F., Lee H.-C., Yeung C.-Y. (2012). Constipation is a major complication after posterior sagittal anorectoplasty for anorectal malformations in children. *Pediatrics and Neonatology*.

[11] Askarpour S., Ostadian N., Javaherizadeh H., Mousavi S.-M. (2014). Outcome of patients with anorectal malformations after posterior sagittal anorectoplasty: a study from Ahvaz, Iran. *Annals of Pediatric Surgery*.

[12] Stenstrom P., Kockum C. C., Emblem R., Arnbjornsson E., Bjornland K. (2014). Bowel symptoms in children with anorectal malformation–a follow-up with a gender and age perspective. *Journal of Pediatric Surgery*.

[13] Borg H. C., Holmdahl G., Gustavsson K., Doroszkiewicz M., Sillen U. (2013). Longitudinal study of bowel function in children with anorectal malformations. *Journal of Pediatric Surgery*.

[14] Kelly J. H. (1972). The clinical and radiological assessment of anal continence in childhood. *ANZ Journal of Surgery*.

[15] Kiesewetter W. B., Chang J. H. (1977). Imperforate anus: a five to thirty year follow-up perspective. *Progress in Pediatric Surgery*.

[16] Stephens F. D. S., Smith E. D. (1986). Classification, identification, and assessment of surgical treatment of anorectal anomalies. *Pediatric Surgery International*.

[17] Ong N.-T., Beasley S. W. (1990). Comparison of clinical methods for the assessment of continence after repair of high anorectal anomalies. *Pediatric Surgery International*.

[18] Holschneider A. M., Jesch N. K., Stragholz E., Pfrommer W. (2002). Surgical methods for anorectal malformations from Rehbein to Pena–critical assessment of score systems and proposal for a new classification. *European Journal of Pediatric Surgery*.

[19] Rintala R. J., Lindahl H. G., Rasanen M. (1997). Do children with repaired low anorectal malformations have normal bowel function?. *Journal of Pediatric Surgery*.

[20] Holschneider A., Hutson J., Pena A. (2005). Preliminary report on the International Conference for the Development of Standards for the Treatment of Anorectal Malformations. *Journal of Pediatric Surgery*.

[21] Pena A. (1995). Anorectal malformations. *Seminars in Pediatric Surgery*.

[22] Brisighelli G., Macchini F., Consonni D., Di Cesare A., Morandi A., Leva E. (2017). Continence after posterior sagittal anorectoplasty for anorectal malformations: comparison of different scores. *Journal of Pediatric Surgery*.

[23] Kyrklund K., Pakarinen M. P., Taskinen S., Rintala R. J. (2015). Bowel function and lower urinary tract symptoms in males with low anorectal malformations: an update of controlled, long-term outcomes. *International Journal of Colorectal Disease*.

